# PCR-Free Detection of Long Non-Coding HOTAIR RNA in Ovarian Cancer Cell Lines and Plasma Samples

**DOI:** 10.3390/cancers12082233

**Published:** 2020-08-10

**Authors:** Narshone Soda, Muhammad Umer, Navid Kashaninejad, Surasak Kasetsirikul, Richard Kline, Carlos Salomon, Nam-Trung Nguyen, Muhammad J. A. Shiddiky

**Affiliations:** 1 School of Environment and Science (ESC), Griffith University, Nathan Campus, QLD 4111, Australia; narshone.soda@griffithuni.edu.au; 2 Queensland Micro-and Nanotechnology Centre (QMNC), Griffith University, Nathan Campus, QLD 4111, Australia; m.umer@griffith.edu.au (M.U.); n.kashaninejad@griffith.edu.au (N.K.); surasak.kasetsirikul@griffithuni.edu.au (S.K.); nam-trung.nguyen@griffith.edu.au (N.-T.N.); 3 School of Engineering and Built Environment, Griffith University, QLD 4222, Australia; 4 Section of Gynecologic Oncology, Ochsner Clinic Foundation, New Orleans, LA 70121, USA; rkline@ochsner.org (R.K.); c.salomongallo@uq.edu.au (C.S.); 5 Exosome Biology Laboratory, Centre for Clinical Diagnostics, University of Queensland Centre for Clinical Research, Royal Brisbane and Women’s Hospital, The University of Queensland, Brisbane, QLD 4029, Australia; 6 Department of Clinical Biochemistry and Immunology, Faculty of Pharmacy, University of Concepción, Concepción 4030000, Chile

**Keywords:** electrochemical detection, HOTAIR RNA, ovarian can cer, naked-eye detection

## Abstract

Long non-coding RNA HOX transcript antisense intergenic RNA (HOTAIR) is one of the promising biomarkers that has widely been used in determining the stages of many cancers, including ovarian cancer. In cancer diagnostics, the two key analytical challenges for detecting long non-coding RNA biomarkers are i) the low concentration levels (nM to fM range) in which they are found and ii) the analytical method where broad dynamic range is required (four to six orders of magnitude) due to the large variation in expression levels for different HOTAIR RNAs. To meet these challenges, we report on a biosensing platform for the visual (colorimetric) estimation and subsequent electrochemical quantification of ovarian-cancer-specific HOTAIR using a screen-printed gold electrode (SPE-Au). Our assay utilizes a two-step strategy that involves (i) magnetic isolation and purification of target HOTAIR sequences and (ii) subsequent detection of isolated sequences using a sandwich hybridization coupled with horseradish peroxidase (HRP)-catalyzed reaction of 3,3′,5,5′-tetramethylbenzidine (TMB) in the presence of hydrogen peroxide. The assay achieved a detection limit of 1.0 fM HOTAIR in spiked buffer samples with excellent reproducibility (% RSD ≤ 5%, for *n* = 3). It was successfully applied to detect HOTAIR in cancer cell lines and a panel of plasma samples derived from patients with ovarian cancer. The analytical performance of the method was validated with standard RT-qPCR. We believe that the proof of concept assay reported here may find potential use in routine clinical settings for the screening of cancer-related lncRNAs.

## 1. Introduction

Genome-wide cancer mutation analyses have revealed an extensive landscape of functional mutations within the non-coding genome, with great emphasis on the expression of long non-coding RNAs (lncRNAs). Long ncRNAs consisting of ∼200 nt or longer transcripts represent a subset of regulatory ncRNAs which are involved in the regulation of post-transcriptional gene expression, epigenomic modulation, and chromatin remodelling, and which have increasingly been identified as key regulators of physiology and pathology [1,2]. Numerous studies have demonstrated the importance of lncRNAs in the identification and management of different types of cancers [3,4,5]. HOX transcript antisense intergenic RNA (HOTAIR) is one of the few well-documented lncRNA that is localized on chromosome 12 within the homeobox C (HOXC) gene cluster [6]. This is a 2.2kb-long transcript that is co-expressed with the HOXC gene cluster and influences gene expression via modulation of chromatin dynamics and epigenetic modifications [6]. Several reports have indicated the aberrant HOTAIR expression in many cancers. Aberrant HOTAIR expression may dysregulate several genes associated with cancer development, and therefore promote the initiation, growth, and invasiveness of tumors [7]. Recent studies have shown that HOTAIR is highly expressed in prostate cancer [8], urothelial carcinoma [9], colorectal cancers [10], gastrointestinal stromal tumors [11], hepatocellular carcinoma [12], pancreatic tumors [13], ovarian cancer tissues [14], and primary breast tumors [7]. The magnitude of HOTAIR expression in primary breast tumors is regarded as an important predictor of patient outcomes such as metastases and death [7,15]. Enforced HOTAIR expression in epithelial cancer cells induces genome-wide retargeting of the polycomb repressive complex 2 (PRC2) and attains a similar pattern to embryonic fibroblasts. This retargeting of the PRC2 complex can result in the alteration of histone H3K27 trimethylation, gene expression, and proliferation of cancer invasiveness and metastases in a manner dependent on PRC2. As a result, HOTAIR has emerged as a promising diagnostic and prognostic biomarker for multiple cancer types.

Ovarian cancer is the seventh most common cancer in women and the eighth leading cause of cancer-related deaths in women worldwide [16]. Due to mild symptoms, ovarian cancer is often diagnosed in the late stage when the tumor has already spread to other parts of the body. As such, early diagnosis and management of ovarian cancer are of the utmost importance. Owing to recent advancements in the development of highly specific gene-amplification and sequencing technologies, more potent and credible cancer biomarkers have been uncovered. Predictive biomarkers such as HOTAIR play a crucial role in guiding treatment decisions and effective individualized therapies. Recently, Yiwei et al. [17] demonstrated that the interaction of HOTAIR and MAPK1 (mitogen-activated protein kinase 1) regulates the proliferation, migration, and invasion of ovarian cancer SKOV3 cells through miR-1, miR-214-3p, and miiR-330-5p, and can serve as a therapeutic target of ovarian cancer. Qui et al. also highlighted the overexpression of HOTAIR in serous ovarian cancer, which correlates with an aggressive tumor phenotype and a poor prognosis [18]. In another study, HOTAIR was demonstrated to enhance the expression of CCND1 and CCD2 by negatively modulating the expression of miR-206 and stimulating the proliferation, cell cycle progression, migration, and invasion of ovarian cancer cells [19]. 

The presence of a low amount of cancer-related lncRNAs in body fluids of early-stage cancer patients presents a significant challenge towards their monitoring. RNA sequencing has been the main method for analyzing lncRNAs in cancer research [20]. Next-generation sequencing (NGS) enables unbiased genome-wide screening and bulk RNA analysis through massive cDNA sequencing [21]. However, this method is tedious and remains costly to be implemented as routine molecular testing. Microarrays are a high throughput method but also use considerable amounts of sample and only report relative amounts of different lncRNAs. These classical methods are relatively robust at the cost of being laboratory-based methods. Amplification-based techniques such as quantitative polymerase chain reaction offer good sensitivity and specificity for RNA detection, but they have drawbacks such as errors due to amplification bias/artifacts. The fluorescence readout also requires costly instruments and fluorescent labels and is susceptible to background fluorescence interference. Therefore, an amplification-free detection methodology could represent an appealing alternative to alleviate these issues. Among many alternative approaches, electrochemical assays have been shown to offer excellent sensitivity and specificity for nucleic acid biomarker detection without any prior amplification process [22,23,24,25]. Additionally, these assays provide intrinsic simplicity, portability, and a high potential for miniaturized, multiplexed, and decentralized analysis of microRNA and other nucleic acid biomarkers with an elevated translational capacity [22,23]. Despite these developments, very few examples of lncRNA biosensors have been reported to date [26,27]. A biosensor with dual readout ability that couples naked-eye visualisation (colorimtric signal) with electrochemical readout is highly compatible for biomolecular sensing, particularly in poor resource environments, where naked-eye evaluation could be used as the first screening pass of the analyte. Over the past years, a few integrated colorimetric and electrochemical assay platforms have been demonstrated for RNA detection [28,29]. Our group has recently developed a simple naked-eye colourimetric and electrochemical assay based on isothermal amplification readout for HOTAIR detection [30]. Although this method has great potential for the development of inexpensive and user-friendly point of care biosensors for resource-poor environments, it relies on isothermal amplification of targets.

In this paper, we report on an amplification-free approach for the naked-eye evaluation and subsequent electrochemical quantification of lncRNA. First, the target lncRNA sequences were magnetically isolated, purified, and released. The released targets were captured by thioloated probe modified screen-printed gold electrodes. The surface-bound lncRNA sequences were then quantified using the horseradish peroxidase (HRP)-modified detection probe. In the detection step, HRP catalyzes the enzymatic oxidation of the 3,3′,5,5′-tetramethylbenzidine (TMB)/H_2_O_2_ system and generates a colored complex to signal the presence of the target lncRNA. As this colored complex is electrochemically active and stable at acidic pH, the amperometric current generated by the complex also quantifies the level of the target lncRNA. This method was first tested on the synthetic lncRNA target spiked in buffer samples and finally demonstrated on cancer cell lines and a panel of plasma samples derived from patients with ovarian cancer.

## 2. Results and Discussion

**Figure 1:**
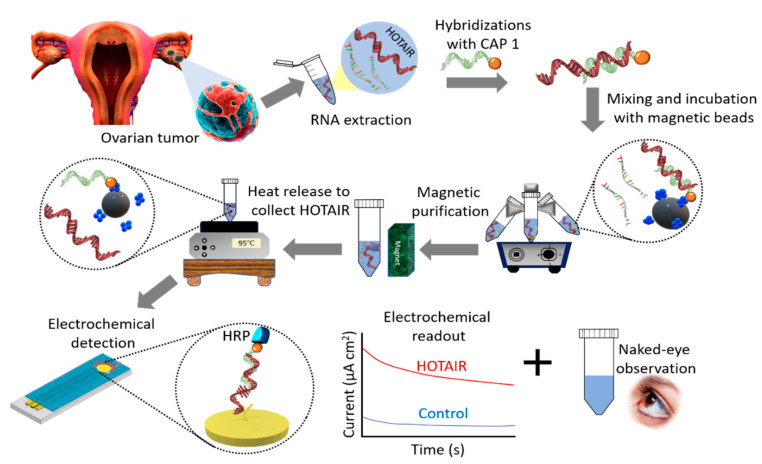
Schematic representation of the amplification free colorimetric detection of lncRNA. Magnetically captured target lncRNAs (top, right) were heat released and adsorbed on a thiolated-DNA modified screen-printed gold electrode (bottom). The relative presence of lncRNA is analyzed by HRP-catalyzed colorimetric reaction in the presence of 3,3′,5,5′-tetramethylbenzidine (TMB)/H_2_O_2_ system.

Figure 1 illustrates the basic principle of HOTAIR detection. Magnetically isolated and purified HOTAIR lncRNA was detected using 3,3′,5,5′-tetramethylbenzidine (TMB)-based colorimetric and electrochemical readouts. Briefly, biotinylated complementary functionalized capture probes attached to streptavidin-coated magnetic beads were dispersed into the sample solution to selectively bind to the HOTAIR strands via the biotin–avidin interactions. Captured HOTAIR targets were then magnetically purified with several magnetic washing steps and released from capture probes through heating. Finally, the released HOTAIR sequences were captured onto self-assembled thiolated capture probes on a modified gold surface of a screen-printed electrode which allowed duplex hybridization with HRP-functionalized detection capture probes. Following the addition of TMB solution, the HRP initiated the oxidation of TMB that produces a blue-coloured charge-transfer complex. This enabled the naked-eye observation of HOTAIR presence. The intensity of the colored complex is proportional to the amount of captured HRP present in the conjugates, which in turn is proportional to the amount of HOTAIR in the RNA sample. The color intensity was also quantified by UV–vis at 652 nm. With the further addition of a stop solution (acid), the blue colored product was converted to a more stable electroactive yellow (diimine) complex, which enabled an alternative amperometric quantification of HOTAIR.

**Figure 2:**
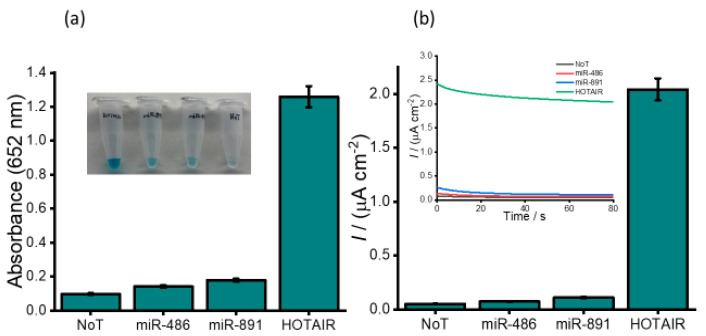
(**a**) Bar diagram corresponding to absorbance obtained for responses for 1.0 pM of miR-486, miR-891 and HOTAIR sequences. (**b**) Bars represent the corresponding current densities for the 1.0 pM of miR-486, miR-891 and HOTAIR sequences. Error bars represent the relative standard deviation of three repeated experiments.

To evaluate the assay functionality, we investigated our assay in the presence and absence of 1.0 pM HOTAIR. The naked-eye observation of the color change showed a subtle color change in the absence of HOTAIR and a profound blue color change in the presence of HOTAIR target. The subsequent UV/visible quantification showed a twelve-fold higher absorbance for HOTAIR compared to the negative control (1.26 HOTAIR vs. 0.098 NoT, no template control) (Figure 2a). Further electrochemical quantification also exhibited a similar profile with a forty-fold higher current density response compared to the negative control (2.04 vs. 0.056 µA cm^−2^), which clearly demonstrates the functionality of our assay. Following the evaluation of the assay functionality, an assessment of the assay selectivity and efficiency of capture probes to isolate HOTAIR lncRNA was performed using non-complementary synthetic sequences of miR-486 and miR-891. Figure 2a inset shows a slight color change for miR-891 and miR-486 which was almost similar to that observed for NoT. A very distinct blue colour was visualized for target HOTAIR. The corresponding absorbance data show a similar trend with an approximately 9-fold higher absorbance of the target HOTAIR compared to non-target (1.26 HOTAIR vs. 0.143 miR-486 and 0.179 miR-891). The subsequent electrochemical quantification (Figure 2b) showed a very small increase in current density response for miR-486 and miR-891 compared to the control data (0.74 and 1.09 vs. 0.056 µA cm^−2^) indicating that our assay is insignificantly affected by non-specific binding of non-target sequences present in the sample. Notably, 1 pM of target HOTAIR exhibited an approximately 40-times higher current density response than that of non-complementary targets (0.39, 0.58 vs. 2.8 µA cm^−2^). The reproducibility of the amperometric measurements for HOTAIR was determined to be a per cent relative standard deviation (% RSD) of 4.32% (*n* = 3), while miR-486 was derived to be 3.78% (*n* = 3), miR-891 was found to be 3.93% (*n* = 3), and no template control was determined to be 3.39% (*n* = 3). These results demonstrate the high specificity of our assay in isolating and subsequent electrochemical detection of HOTAIR.

### 2.1. Detection of HOTAIR lncRNA in Spiked Buffer Samples

**Figure 3:**
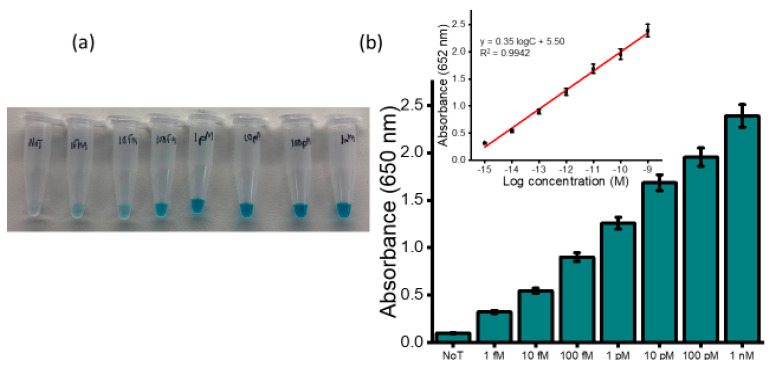
Sensitivity of the assay. (**a**) Picture of the naked-eye detection of HOTAIR derived from a series of synthetic lncRNA sequences (1 fM-1 nM) (left panel); (**b**) the corresponding bar diagram for absorbance at 652 nm (right panel). The inset shows the analogous calibration plot with error bars representing the standard deviation of three independent experiments.

**Figure 4:**
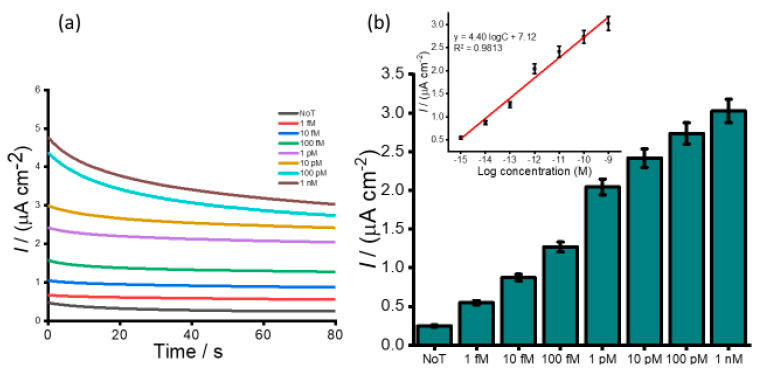
Corresponding i–t curve (left panel) (**a**) and bar diagram (right panel) (**b**) for the amperometric current density obtained from a series of HOTAIR targets (1fM-1 nM). The inset depicts the calibration plot with error bars representing the standard deviation of three independent experiments.

To assess the dynamic range for detection of HOTAIR lncRNA, serial dilutions of HOTAIR sample were measured ranging from 1 fM to 1 nM. A gradual increase in the color intensity as the concentration increase was visually observed (Figure 3a). The intensity of the color obtained for 1 fM was clearly distinguished from that of the NoT. The subsequent UV/vis quantification of the color changes produced a similar increasing profile of absorbance values as the color intensity corresponding to the target concentration increases (Figure 3b). Following the addition of an acid, the electrochemical measurement using amperometry resulted in increased signals quantitatively with RNA concentration (Figure 4a). The calibration plot in Figure 4b (inset) showed good linearity from 1 fM to 1 nM of HOTAIR lncRNA with a correlation coefficient, R^2^, of 0.9813. From the current density responses, detection of the target RNA above background was confirmed down to 1 fM which was easily distinguishable from the control (0.49 µA cm^−2^ NoT vs. 1.22 µA cm^−2^ 1 pM HOTAIR). A relative standard deviation for three independent measurements was calculated to be < 5.0%, indicating the excellent reproducibility of the assay towards the quantification of HOTAIR lncRNA. Our femtomolar detection limit was comparable to previously reported electrochemical assays for lncRNA [28,29]. Our previously reported isothermal amplification-based method for electrochemical quantification of HOTAIR also offers similar LOD [30]. However, the key benefits of our current assay are that it provides a novel amplification-free detection of HOTAIR species using a single technique. Hence, it avoids possible degradation of HOTAIR target as a result of amplification or target modifications commonly associated with conventional assays.

### 2.2. Detection of HOTAIR lncRNA in Spiked Plasma Samples

**Figure 5:**
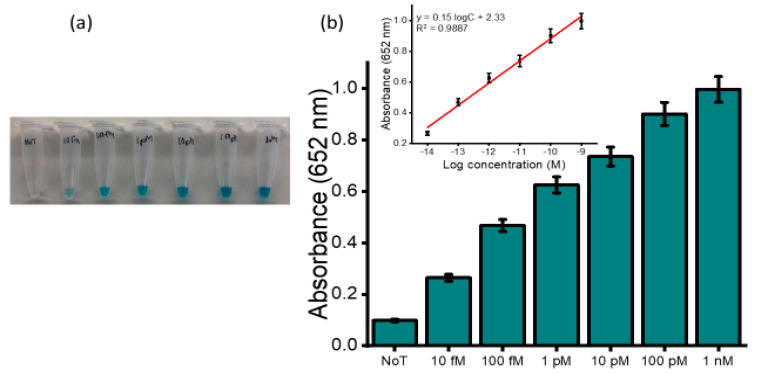
(**a**) Picture of the naked-eye detection corresponding to the designated starting concentration of HOTAIR sequences ranging from 10 fM to 1.0 nM in spiked plasma samples. (**b**) Corresponding bar diagram of the absorbance at 652 nm for the designated concentrations of HOTAIR. Inset, linear calibration plot showing concentration–current density relationship. Error bars represent the standard deviation of three repeated experiments.

**Figure 6:**
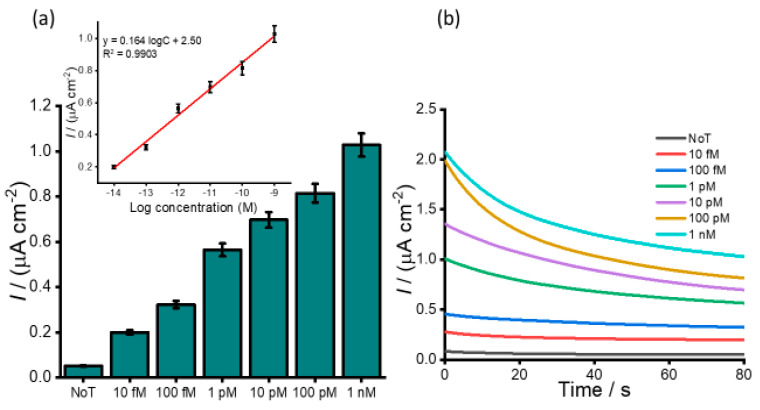
(**a**) Bar diagram corresponding current densities for the designated concentrations of spiked plasma. Inset, linear calibration plot showing concentration–current density relationship. (**b**) Amperometric responses corresponding to the designated starting concentration of HOTAIR sequences ranging from 10 fM to 1.0 nM in spiked plasma samples. Error bars represent the standard deviation of three repeated experiments.

Our developed assay was further examined for its specificity and sensitivity in biological fluids. The analysis was performed in pretreated and undiluted healthy human plasma spiked with varying concentrations of synthetic HOTAIR sequences. Figure 5a depicts the naked-eye observation of the color intensities corresponding to the spiked concentrations. An increase in color intensity as the varying target concentration increase was visually observed. UV measurements were further conducted to quantify the obtained colored complex (Figure 5b) at an absorbance of 650 nm and consistently produced absorbance that was incremental as the concentration increased with a relative standard deviation (%RSD) ≤5%, *n* = 3). The limit of detection was determined to be 10 fM when the blue coloured complex was still visibly distinguished to that of the no template control with a correlation coefficient (R^2^) of 0.9887 (y = 0.15 logC + 2.33, where C is the concentration) (inset). The subsequent electrochemical measurement obtained for the serially diluted target exhibited an increase in the current density response for the varying spiked samples with an increase in concentrations of the target, as shown in Figure 6a. This illustrates the potential applicability of the assay to analyze complex serum samples. Figure 6b demonstrates the amperometric responses corresponding to the designated starting concentration of HOTAIR sequences ranging from 10 fM to 1.0 nM in spiked plasma samples. The amperometric current density measurement estimated an LOD of 10 fM with a linear dynamic range between 10 fM to 1 nM. The reproducibility of the serial spiked concentrations was evaluated using three independent electrodes and produced an RSD of <5% between the electrodes for each concentration. It is worth highlighting that we observed a tenfold decrease in sensitivity of the spiked plasma sample compared to that of the buffer. This change may be attributed to the complexity of the plasma sample, which contains millions of other biomolecules that may have the potential to interfere nonspecifically with our target. In addition, non-specific binding during the magnetic separation and purification step, as well as non-specific adsorption in the detection process, could have potentially contributed to the decrease in the sensitivity. The detection limit of the assay is comparable to other existing electrochemical methods for lncRNAs detection [31,32].

### 2.3. Detection of lncRNA in Ovarian Cancer Cell Line Samples

**Figure 7:**
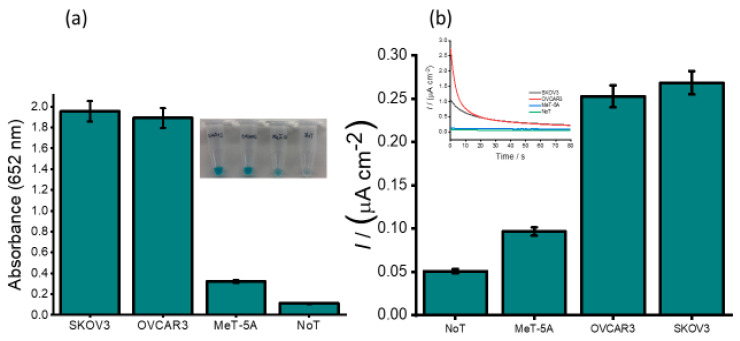
Analysis of ovarian cancer and normal cell lines. (**a**) Absorbance (UV–vis) obtained for SKOV3, OVCAR3 (ovarian cancer) and MeT-5A (non-cancerous) cell lines, no-template (NoT) controls (inset: picture of the naked-eye detection). (**b**) Amperometric responses for HOTAIR sequences in cell lines. Error bars represent the standard deviation of three repeated experiments.

To determine whether our developed method can be used in real biological samples, we examined our assay in the total RNA sample extracted from human ovarian cell lines (SKOV3 and OVCAR3) and one non-cancerous cell line (Met-5A) as a control. As shown in Figure 7a, the picture of the naked-eye visualization of the color changes corresponding to the cell lines. A faint blue colour corresponding to the non-cancerous cell line (Met5A) was observed, and the positive ovarian cancer cell lines (SKOV3 and OVCAR3) showed a more pronounced blue color. A 6-times higher absorbance values were obtained for the positive ovarian cancer cell lines compared to that of the non-cancerous cell line. The corresponding bar diagram for SKOV3 and OVCAR3 and typical amperometric curves (Figure 7b) demonstrated that the current density response obtained with the ovarian cancer cell lines SKOV3 and OVCAR3 was significantly higher than that of the normal cell line (0.269 and 0.253 vs. 0.097 µA cm^−2^). This suggests that HOTAIR lncRNA is overexpressed in SKOV3 and OVCAR3 compared to the normal cell line. Compared to previously reported data, our results were in good agreement showing a similar profile for HOTAIR expression levels [33]. Our assay showed good reproducibility (%RSD of <3.94% for *n* = 3) for the interassay signals. We then validated our assay performance in cancer cells using RT-QPCR. A similar profile to our assay was observed, which strongly supports our findings. This demonstrates that the electrochemical signals generated with our assay could potentially determine the presence or absence of HOTAIR in cell lines. This demonstrates that our developed method may aid in the detection of expression levels of HOTAIR in cells obtained from cancerous patients.

### 2.4. Detection of lncRNA in Ovarian Cancer Patient Samples

**Figure 8:**
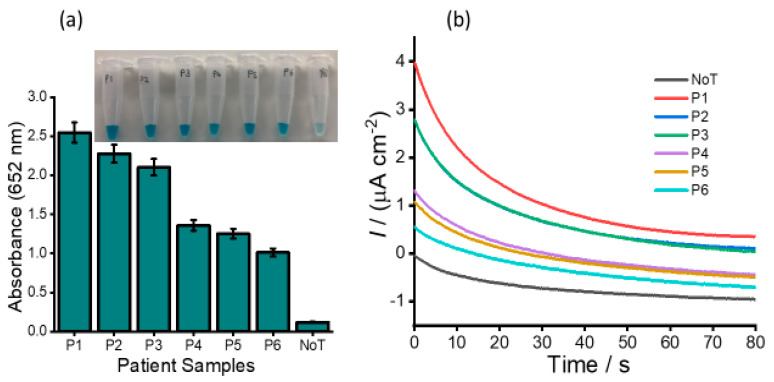
Analysis of human serum patient samples. (**a**) Bar diagram representing the absorbance for six serum samples collected from six ovarian cancer patients (P1, P2, P3 = high-grade serous subtypes and P4, P5, P6 = benign). (Inset: picture of the naked-eye screening); (**b**) Corresponding amperometric responses for RNA extracted from these six samples. For comparison, no template control (NoT) data are used. Error bars represent the standard deviation of three repeated experiments.

The expression and clinical values of HOTAIR lncRNA in ovarian cancer patients were further examined using human serum samples of six newly diagnosed patients. The selected samples comprised three ovarian cancer high-grade serous subtypes (P1, P2 and P3) and three benign samples (P4, P5 and P6), Figure 8. Our assay detected all the cancer samples to be HOTAIR positive, and the absorbance value of ovarian cancer high-grade serous subtype was approximately 20-times higher (absorbance at 652 nm = 2.557/2.289/2.114 vs. 0.117). The amperometric signals generated by the assay could also distinguish different HOTAIR levels in clinical samples, as indicated in Figure 8b**.** The high-grade epithelial ovarian positive samples showed at least a 2-times higher current density response as compared to benign samples, which suggests an upregulation of HOTAIR in ovarian cancer patients. The clinical data show good reproducibility of our assay (%RSD ≤ 5%, for *n* = 3) for analyzing expression profiles of HOTAIR in different stages of ovarian cancers. Thus, the developed assay can directly measure HOTAIR expression levels in human serum without prior amplification or pretreatment and provide great potential in clinical diagnosis.

The naked-eye discrimination analysis demonstrated here holds great promise for the development of low-cost and user-friendly point-of-care biosensors for resource-poor environments. The dual biosensing technique employed here incorporates visual identification, which can be used as a first-pass screening for ovarian cancer followed by UV–visible or electrochemical detection, which could be used to quantify the amount and gravity of the disease. Our amplification-free assay relies on the use of disposable and cost-effective screen printed electrodes (<$4.0 AUD per electrode) which also helps to minimize the non-specific response that often arises from numerous surface reactions associated with conventional disk electrodes. Furthermore, the avoidance of time-consuming cleaning procedures reduces the assay time and allows miniaturized and decentralized analysis of HOTAIR, especially in resource-limited environments. Another beneficial aspect of our assay is that the magnetic separation technique provides rapid and efficient isolation and purification of HOTAIR target and increases the assay performances by reducing the matrix effects of biological samples. The use of dual hybridization enables high specificity, which subsequently improves the sensitivity. Thus, the overall analytical performance of our developed assay (1 fM LOD with excellent reproducibility (%RSD ≤ 5 % for *n* = 3) demonstrates its high potential for miniaturized and decentralized analysis of RNA biomarkers with increased translational capacity.

Although our assay exhibits excellent performance, it has several limitations. One of the major drawbacks limiting the sensitivity of our assay is the non-specific adsorption of the signaling probe, and enzyme conjugates integrated for detection. This results in a significant background response. Furthermore, the fabrication process requires several steps, which are time-consuming and thus complicate the assay. In addition, the folding tendency of lncRNAs into various secondary or tertiary structures when immobilized on the electrode surface reduces their structural stability. This issue subsequently decreases the analytical performance of the assay. Another key aspect is the sensing platform architecture which determines the target surface coverage and intensity of non-specific interactions. However, many efforts have recently been directed towards the use of aromatic thiols to facilitate monolayer formation. This significantly improves the sensitivity of the assay.

Despite the significant progress made in sandwich hybridization sensors, key design aspects determining the performance of these sensors still need to be addressed before they can be translated into portable point-of-care devices. Major aspects of development include probe design, which is essential for the overall selectivity and sensitivity of the assay. Another key element of development is the development of innovative strategies that could integrate the isolation, purification, immobilization, and detection steps in a single device, which could be vital in routine clinical applications. More so, the coupling of these devices with amplification techniques in automated platforms would pave the way for accurate detection systems.

## 3. Materials and Methods

### 3.1. Reagents and Chemicals

All the reagents and chemicals used in this study were of analytical grade and purchased from Sigma Aldrich (Sydney, NSW, Australia). UltraPureTM DNase/RNase-free distilled water (Invitrogen, Australia) was used throughout the experiments. Synthetic lncRNA and capture probes were purchased from Integrated DNA Technologies (Coralville, Iowa USA) and sequences are shown in Table 1. Hydroquinone and hydrogen peroxide were obtained from Sigma Aldrich (Sydney, New South Wales, Australia). SPE-Au (DRP-C220 AT) was purchased from Metrohm Dropsens (Oviedo, Spain).

### 3.2. Preparation of RNA from Cell Line and Ovarian Cancer Samples

RPMI-1640 growth medium (Life Technologies, Victoria, Australia) supplemented with 10% fetal bovine serum (Life Technologies, Australia) and 1% penicillin/streptomycin (Life Technologies) was used to culture ovarian cancer cell lines (SKOV3 and OVCAR3) and a non-cancerous cell line (Met-5A). These cells were cultured in a humidified incubator with 5% CO_2_ flow at 37 °C. All cells were harvested by standard trypsinization protocol after they reached 70–80% confluence. Briefly, cells were washed with 3–5 mL HBSS (Gibco, Victoria, Australia) to remove enzyme inhibitors followed by 1–2 mL of TryPLe (Gibco) and incubation for 3 min at 37 °C. To neutralize the trypsin activity, of cell culture media 1 × 4 volumes TryPLe was added, followed by centrifugation for 5 min at 2500 rpm. A cell pellet was washed with PBS and centrifuged for 5 min at 2500 rpm. Then, the cell pellet was collected for RNA extraction and stored at –20 °C until further processing. Plasma samples were collected according to the declaration of Helsinki and approved by the Ethics Committee of the University of Queensland (approval number 2016000300) and the Ochsner Medical Center (New Orleans, Louisiana, USA). Plasma was isolated from the whole blood sample by centrifuging at 2000× *g* for 10 min and was stored at −80 °C until analysis. Ovarian cancer samples were collected accordingly, assigned, and classified based on their histotype (e.g., stage I and stage III), and stored at −80 °C in the Biobank units. In this study, only patients with epithelial ovarian cancer high-grade serous subtype (*n* = 3) and benign samples (*n* = 3) were utilized. miRNeasy Mini Kit (Qiagen, Victoria, Australia) was used to extract RNA, and the concentration was quantified using a SPECTROstar Nano Microplate Reader (BMG Labtech) operated by MARS data analysis software.

### 3.3. Magnetic Isolation and Purification of HOTAIR lncRNA Target

The target-specific biotinylated capture probe 1 (CP1) was mixed with 10 μL of synthetic HOTAIR lncRNA, 10 μL of 5 × SSC buffer (pH 7.0), and 15 μL of 10 μM biotinylated CP1 (Probe 1 Table 1). The mixture solution was heated at 55 °C for two minutes and placed on a thermomixer (300 rpm) for 1 h at room temperature (25 °C) to allow hybridization of CP1 with target HOTAIR lncRNA. Next, 20 μL of streptavidin-coated Dynabeads^®^ (MyOne Streptavidin C1, Invitrogen, Victoria, Australia) was washed three times with 2 × binding and washing (B&W) buffer (10 mM Tris-HCl, pH 7.5; 1.0 mM EDTA; 2.0 M NaCl) and resuspended in 20 μL of 2X B&W buffer. The beads were dispersed into the solution containing CP1/target lncRNA complex and were incubated for 30 min at 25 °C to allow the formation of beads/CP1/target lncRNA complex. The beads/CP1/target lncRNA complex was separated using a magnet, washed three times with 2 × B&W buffer, and resuspended in 9.0 μL of RNase-free water. The magnetically captured isolates were heated for two minutes at 95 °C, and we immediately collected the heat-released target HOTAIR lncRNA from the supernatant using an external magnet. A total of 5.0 μL of the released HOTAIR lncRNA was diluted with 15 μL of 5 × SSC buffer (pH 7.0) and used for naked-eye visualization and electrochemical quantification.

### 3.4. Fabrication of Sensor for UV-Vis and Electrochemical Analysis

Electrochemical measurements were performed using a CH1040C potentiostat (CH Instruments, Austin, Texas, USA) on screen-printed gold electrodes (SP-Au), which contained a three-electrode system (gold working, Ag/AgCl reference and gold counter electrode). Prior to probe adsorption, the effective working area of each electrode was measured as a function of scan rate under cyclic voltammetric conditions for the one-electron reduction of [Fe(CN)_6_]^3−^ using the Randles–Sevcik equation,

(1) $$i_{p}=\left(269\times 10^{5}\right)\;n^{3/2}A\;D^{1/2}\;C\;\nu^{1/2}$$

where $i_{p}$ is the peak current (*A*), *n* is the number of electrons transferred (Fe^3+^ → Fe^2+^, *n* = 1), *A* is the effective area of the electrode (cm^2^), *D* is the diffusion coefficient of [Fe(CN)6]^3-^ (taken to be 7.60 × 10^−5^ cm^2^s^−1^), *C* is the concentration (mol cm^−3^), *ν* is the scan rate (Vs^−1^). Differential pulse voltammetric (DPV) experiments were recorded in 10 mM PBS solution containing a 2 mM [K_3_Fe(CN)_6_] electrolyte solution at a potential range of −0.2 V to 0.5 V, with a pulse amplitude of 50 mV, and a pulse width of 50 ms to characterize the stepwise sensor fabrication process.

Then, 10 µL of 10 µM thiolated capture probe (CP2, Table 1) was mixed with 20 µL of 10 mM tris(2 carboxyethyl)phosphine (TCEP) and incubated for 1 h in the dark to reduce the disulphide bond of the capture probe. This was followed by the addition of 70 µL of 1 × PBS (pH 7.4) to the mixture and subsequent immobilization onto bare SP-Au surface (5 µL) then incubation for 2 h to form the self-assembled monolayer of CP2 on SP-Au surface. Following incubation, 2 mM of 6-mercaptohexanol solution was added to the electrode and incubated for 3 h in the dark at room temperature. Then, 5 µL of previously released HOTAIR lncRNA target was hybridized with the surface-bound CP2 for 30 min on a thermomixer (300 rpm). Further hybridization with 5 µL of biotinylated detection probe (DP) for 30 min was performed to allow duplex formation. By taking advantage of the biotin-streptavidin affinity interactions, 5 µL of 10 ng/µL streptavidin-conjugated HRP was immobilized on the modified electrode surface for 30 min. Each of the fabrication steps was performed at 25 °C and followed by a washing step with 1 × PBS pH 7.4. Then, 50 uL of TMB solution was added and incubated for 5 min in the dark. The color change was also quantified by UV–vis at 652 nm. With the further addition of 1.0 µL stop solution (acid), the blue colored product was converted to a more stable electroactive yellow (diimine) complex, which facilitated amperometric measurements at 150 mV for 120 s.

## 4. Conclusions

In conclusion, we developed a PCR-free diagnosis assay based on simple colorimetric observation and electrochemical detection for sensitive and specific detection of HOTAIR in ovarian cancer. Our method achieved a high analytical performance (i.e., sensitivity (LOD = 1 fM, reproducibility, %RSD ≤ 5.0), dynamic range (1 pM–1 nM)) in analyzing HOTAIR expression levels derived from human cancer cells and serum samples obtained from ovarian cancer patients. The developed assay offers several benefits such as efficient target separation and purification, minimum matrix effects and reduced assay time, as well as user-friendly and cost-effective device construction for point-of-care applications. Our assay has potential applications in cancer screening and prognosis, and assists in targeted therapies and personalized treatments.

## Figures and Tables

**Table 1:** Oligonucleotide sequences used in experiments.

Oligos	5′-Sequences-3′
HOTAIR Cap 1	ATC AAT TAA TTA GCG CCT CCC AGT CCC /3bio
HOTAIR Cap 2	5ThiolMC6-D/ACG CCG CCA TAT TTT ACA GTC CAA AGG A
HOTAIR Synth	GGG ACU GGG AGG CGC UAA UUA UAA AAU AUG GCG GCG U
miR-891 Synth	UGC AAC GAA CCU GAG CCA CUG A
miR-486 Synth	UCC UGU ACU GAG CUG CCC CGA G
